# Making CAR T Cells a Solid Option for Solid Tumors

**DOI:** 10.3389/fimmu.2018.02593

**Published:** 2018-11-08

**Authors:** Andrea Schmidts, Marcela V. Maus

**Affiliations:** Cellular Immunotherapy Program, Cancer Center, Massachusetts General Hospital, Harvard Medical School, Boston, MA, United States

**Keywords:** immunotherapy, CAR-T cells, solid tumors, cancer, toxicity, cell engineering

## Abstract

Adoptive cell therapy with chimeric antigen receptor (CAR) T cells aims to redirect the patient's own immune system to selectively attack cancer cells. To do so, CAR T cells are endowed with specific antigen recognition moieties fused to signaling and costimulatory domains. While this approach has shown great success for the treatment of B cell malignancies, response rates among patients with solid cancers are less favorable. The major challenges for CAR T cell immunotherapy in solid cancers are the identification of unique tumor target antigens, as well as improving CAR T cell trafficking to and expansion at the tumor site. This review focuses on combinatorial antigen targeting, regional delivery and approaches to improve CAR T cell persistence in the face of a hostile tumor microenvironment.

## Introduction

**Figure 1:**
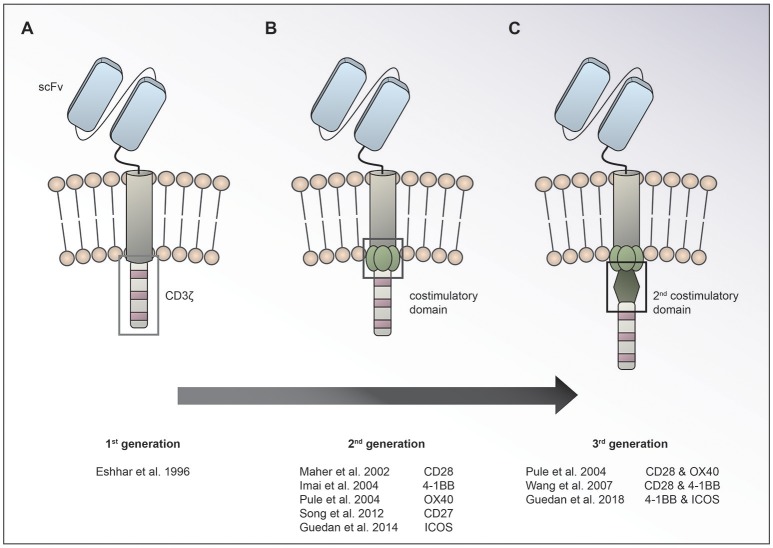
Evolution of CAR design. The basic CAR set up consists of an antigen binding moiety (e.g., scFv based) and a spacer on the extracellular side, a transmembrane domain and domains for T cell activation on the intracellular side. While 1st generation CARs **(A)** contain only a CD3ζ chain for T cell activation, 2nd **(B)**, and 3rd **(C)** generation CARs have one or two costimulatory domains incorporated, respectively.

**Figure 2:**
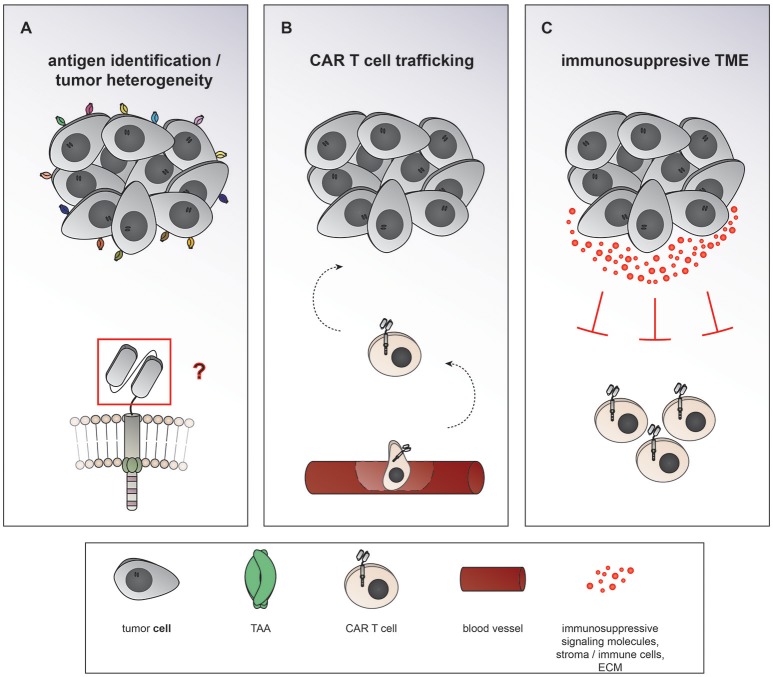
Major hurdles to the efficiency of CAR T cells in solid cancers. **(A)** Heterogeneous expression of tumor associated antigens (TAA) on solid cancers as well as overlapping expression on healthy tissues makes it difficult to find suitable targets of CAR T cells therapy. **(B)** After intravenous application CAR T cells need to traffic to the tumor site, extravasate the circulation, and penetrate the tumor. **(C)** The term tumor microenvironment describes the interplay between the tumor cells themselves and the surrounding blood vessels, stromal cells, immune cells, as well as the extracellular matrix. CAR T cell migration and expansion are inhibited by the immunosuppressive environment of solid cancers.

Chimeric antigen receptor (CAR) T cells targeting CD19 for the treatment of relapsed/refractory (r/r) B-cell acute lymphoblastic leukemia (ALL) and lymphoma have led to unprecedented response rates of about 80% in a patient population that up until then had a very poor prognosis (1–7). The FDA approval of CAR T cells for leukemia and then lymphoma in 2017 marked the breakthrough of two converging clinical research fields: CAR T cell immunotherapy and gene therapy. CAR T cells were first conceived by Eshhar and colleagues in 1989 as an enhanced T cell version endowed with an antibody-based recognition domain fused to a CD3zeta signaling domain (Figure 1A) (8). Over the years, these so called first generation CAR T cells have experienced an improvement of their anti-tumor potency by adding one (9–13) or two costimulatory domains (11, 14, 15), resulting in second or third generation CAR T cells, respectively (Figures 1B,C). Making the impressive potency of CAR T cell therapy available to the more numerous patients suffering from solid cancers has been an endeavor for about a decade now. So far, CAR T cells for solid tumors have not been able to achieve the impressive responses induced in hematological cancers. Identification of unique tumor associated antigens (TAA), CAR T cell trafficking and persistence, as well as the immunosuppressive tumor microenvironment have emerged as the major drawbacks to the success of CAR T cells for the treatment of solid malignancies (Figure 2). Multiple approaches aiming at overcoming these hurdles are currently under active investigation.

## Status of clinical research on CAR T cells for solid tumors

### Most promising results of CAR T cell trials for solid tumors so far

Recapitulating the history of chemotherapy, CAR T cells for the treatment of solid cancers have not yet been able to reproduce the success of their hematological counterparts. Nevertheless, the field has achieved important breakthroughs in the treatment of some solid tumors. In a phase I clinical trial investigating GD2 specific CAR T cells for the treatment of pediatric neuroblastoma, 3 out of 11 patients who had active disease at the time of enrollment achieved a complete remission (16). Encouraging results were also reported from a phase I/II clinical study using a HER2 specific CAR in patients with HER2-positive sarcoma. Of the 17 evaluable patients, all of whom had relapsed or refractory disease, 3 had stable disease and were able to undergo surgery to remove the residual tumor, resulting in complete remission without further treatment (17). In a study conducted by Brown and colleagues, regional, multi-dose treatment with IL13Rα2 specific CAR T cells induced a complete remission in a patient with disseminated glioblastoma (18).

### Current CAR T cell clinical trials for solid tumors

There are currently over 270 CAR T cell trials registered at the U.S. National Library of Medicine (ClinicalTrials.gov). Of these, about one third are investigating the use of CAR T cells for solid tumor indications. Table 1 shows selected CAR T cell trials for solid cancers that are currently recruiting patients in the US and Europe. Among the most studied solid tumor targets are EGFRvIII for glioblastoma (NCT03283631, NCT02664363, NCT01454596), GD2 for neuroblastoma (NCT03373097, NCT03294954, NCT02761915) and mesothelin for various epithelial cancers (NCT02792114, NCT01583686). Interestingly, several trials are exploring regional delivery routes of CAR T cell therapy, especially intracranial administration for glioblastoma and other brain tumors (NCT03283631, NCT03500991, NCT01818323, NCT02208362).

**Table 1:** Selected CAR T cell trials for solid tumors.

	**Indication**	**Lympho-depletion**	**Route of administration**	**Distinctive features**	**Identifier**	**Center**
CD70	Pancreatic/Renal Cell/Breast/Ovarian Cancer, Melanoma	Cyc, Flu	Systemic	IL-2 administration	NCT02830724	NCI
CD171	Neuroblastoma	N/S	Systemic	tEGFR	NCT02311621	Seattle Children's Hospital
EGFRvIII	Recurrent Glioblastoma/-sarcoma	–	Intracerebral	radiolabeling (111In) of CAR T cells	NCT03283631	Duke
	Glioblastoma/-sarcoma	TZM	Systemic	radiolabeling (111In) of CAR T cells	NCT02664363	Duke
	Glioblastoma/-sarcoma, Brain Cancer	Cyc, Flu	Systemic	IL-2 administration	NCT01454596	NCI
ErbB	Head and Neck Cancer	–	Intratumoral	–	NCT01818323	King's College London
FAP	Malignant Pleural Mesothelioma	–	Intrapleural	–	NCT01722149	University Hospital Zurich
GD2	(r/r) Neuroblastoma	N/S	Systemic	iCas9	NCT03373097	Bambino Gesù Hospital, Rome
	Neuroblastoma	Cyc, Flu	Systemic	NK T cells, IL-15 administration	NCT03294954	Texas Children's Hospital
	r/r Neuroblastoma	Cyc, Flu	Systemic	–	NCT02761915	UCL, Great Ormond Street Hospital for Children
GPC3	Pediatric Solid Tumors	Cyc, Flu	Systemic	–	NCT02932956	Texas Children's Hospital
	Hepatocellular Carcinoma	Cyc, Flu	Systemic	–	NCT02905188	Houston Methodist Hospital
HER2(ErbB2)	r/r Pediatric CNS Tumors	–	Intracerebral	tEGFR	NCT03500991	Seattle Children's Hospital
	r/r Glioblastoma	–	Intracerebral	tCD19	NCT03389230	City of Hope Medical Center
	r/r Glioblastoma	–	Intracerebral	–	NCT02442297	Houston Methodist Hospital
	Sarcoma	– / Flu / Cyc, Flu	Systemic	–	NCT00902044	Houston Methodist Hospital
IL13Rα2	Glioblastoma, r/r Brain Neoplasm	–	Intracerebral	tCD19	NCT02208362	City of Hope Medical Center
MET	Melanoma, Breast Cancer	–	Systemic	CAR transfer by RNA electroporation	NCT03060356	UPenn
Mesothelin	Breast Cancer	Cyc	Systemic	–	NCT02792114	MSKCC
	Cervical/Pancreatic/Ovarian/Lung Cancer, Mesothelioma	Cyc, Flu	Systemic	IL-2 administration	NCT01583686	NCI
MUC-16 (ecto)	Recurrent Ovarian/Primary Peritoneal/Fallopian Tube Carcinoma	Cyc, Flu	Systemic and intraperitoneally	IL-12 secreting,tEGFR	NCT02498912	MSKCC
PSCA	Prostate Cancer	–/Cyclo	Systemic	TGF-β resistant CAR T cells	NCT03089203	UPenn
	Pancreatic Cancer	N/S	Systemic	Rimiducid inducible costimulation	NCT02744287	Baylor Sammons Cancer Center
ROR1	Triple Negative Breast Cancer, NSCLC	Cyc, Flu	Systemic	–	NCT02706392	Fred Hutchinson Cancer Research Center

*CD, cluster of differentiation; Cyc, cyclophosphamide; EGFRvIII, epidermal growth factor receptor vIII; ErbB, erythroblastosis oncogene B; FAP, fibroblast activation protein alpha; Flu, fludarabine; GD2, disialoganglioside; GPC3, glypican 3; HER2, human epidermal growth factor receptor 2; iCas9, inducible caspase-9 (safety switch); IL13Rα2, Interleukin-13 receptor subunit alpha-2; MET, tyrosine-protein kinase MET (mesenchymal to epithelial transition); MUC-16(ecto), extracellular portion of the glycosylated mucin, MUC16; N/S, not specified; NSCLC, non-small-cell lung cancer; PSCA, prostate stem cell antigen; ROR1, receptor tyrosine kinase like orphan receptor 1; r/r, relapsed/refractory; tCD19/tEGFR, truncated CD19 / EGFR (safety switch); TGF-β, Transforming growth factor beta; TZM, Temozolomide*.

## Finding the right target antigens

The ideal target epitope for CAR T cell therapy would be expressed on every tumor cell and crucial for the maintenance and propagation of the malignant phenotype, while being absent on healthy tissues. In practice, the identification of target antigens that are solely present on malignant- but not on healthy cells- has proven rare. Finding suitable TAA has been easier for hematological malignancies than for solid tumors. On-target, off-tumor toxicities of CD19 and BCMA specific CARs, in the form of B cell and plasma cell aplasia, are usually manageable in patients with hematological malignancies. In contrast, on-target, off-tumor toxicities of CARs for solid cancers can lead to fatal outcomes (19). Potential reasons for this may include overlapping antigen expression on epithelial tissues, which most solid tumors originate from, and the spatial confinement of critical sites when targeting solid tumors. Target antigen density on the tumor cells has been shown to positively correlate with CAR T cell functionality, evidenced by activation and cytokine production (20, 21). Thus, finding a target molecule that is highly expressed on the tumor cells is desirable for two reasons- to enhance CAR T cell potency and to avoid on-target, off-tumor toxicities to healthy tissues expressing the target antigen at low levels.

### Combinatorial antigen targeting

Heterogeneous antigen expression on solid tumors as well as low-level expression of TAA on healthy tissues render it difficult to find well suited targets for CAR T cell therapy of solid tumors. Combinatorial antigen recognition approaches have recently been developed to address these challenges.

#### “OR” gate/tandem CAR

**Figure 3:**
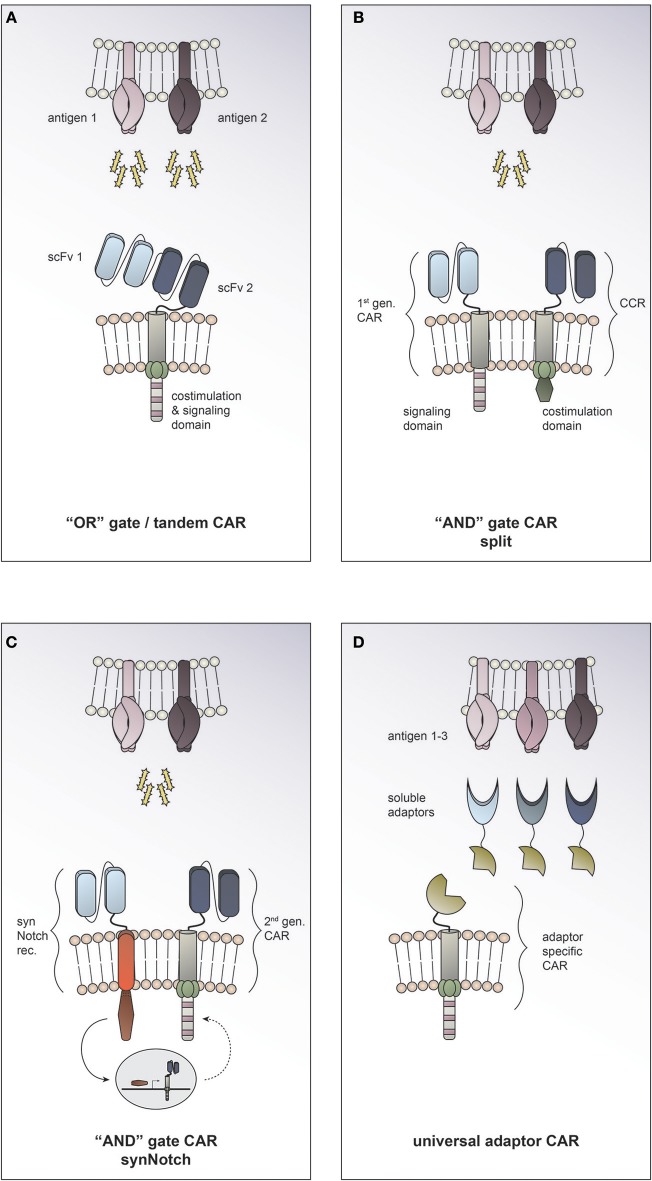
Combinatorial antigen targeting for solid cancers. **(A)** For “OR” gate/ tandem CAR T cells the presence of one antigen is sufficient to trigger effector function, while concurrent expression of both antigens leads to synergistical improvement of activation. **(B**+**C)** “AND” gate CAR T cells require the presence of either target antigens to efficiently activate. **(B)** The split CAR approach taken by Kloss and colleagues uses a 1st generation CAR that recognizes antigen 1 combined with a chimeric costimulatory receptor (CCR) that provides the necessary costimulation upon encounter of antigen 2. **(C)** In the synthetic Notch (synNotch) approach reported by Roybal and colleagues sensing of antigen 1 by a synNotch transcriptional receptor (synNotch rec.) induces expression of a CAR that is specific for antigen 2. **(D)** Universal CAR T cells can target a variety of different antigens since their antigen specificity comes from the administration of soluble adaptors.

Employing Boolean “OR” logic allows targeting two or more TAAs with a single CAR T cell. In so called tandem CARs the presence of either antigen 1 or antigen 2 is enough to trigger activation (Figure 3A). This strategy helps to increase the density of the targetable molecules on the tumor surface and therefore may increase CAR T cell potency. In tandem CAR T cells, effector function is synergistically improved upon co-recognition of both target antigens, while it is still preserved in the presence of only one antigen. Indeed, enhanced antitumor efficacy of dual antigen targeting has been reported in preclinical models for solid and hematological cancers (22–24). Hedge and colleagues designed a HER2/IL13Rα2 tandem CAR for the treatment of glioblastoma. They found the activation characteristics of the HER2/IL13Rα2 tandem CAR to be comparable to those of the corresponding single antigen specificity CAR in the presence of one target antigen. However, when both target molecules were expressed concurrently, heterodimers were induced and a synergistic effect on the CAR T cell activation was observed. Compared to the single antigen specificity CAR T cells, the tandem CAR could delay tumor growth, mitigate antigen escape and improve survival in a glioblastoma mouse model (23). To date it is unclear how the toxicity profile of tandem CAR T cells compares clinically to single antigen specificity CAR T cells. On the one hand, it has been suggested that by endowing tandem CAR T cells with reactivity against two TAA they may display an improved ability to discriminate malignant vs. normal target cells. On the other hand, prediction of potential on-target, off-tumor toxicity sites is rendered more complex, since expression of each of the targeted molecules individually and in combination must be taken into consideration. There is an open phase I clinical trial using a tandem CAR directed against CD19 and CD20 for patients with relapsed/refractory B cell malignancies (NCT03019055). To date, no tandem CAR trials for solid tumors have been opened (according to clinicaltrials.gov).

#### “AND” gate CAR

Employing Boolean “AND” logic, CAR T cells can be reprogrammed to activate only in response to target cells expressing two antigens concurrently (Figure 3B+C); thereby allowing them to discriminate more safely between malignant cells and healthy tissues. This can be achieved by engineering T cells to express both, a first-generation CAR that recognizes antigen 1 but induces only inadequate activation, and a chimeric costimulatory receptor that recognizes antigen 2 and allows for full T cell activation by complementing the co-stimulation needed. Kloss and colleagues provided proof of concept that an “AND” gate to regulate CAR T cell activity can be generated by re-associating signal 1 and signal 2 and applied this in the context of a prostate tumor model using PSMA and PSCA antigens (25) (Figure 3B). A different approach to generating “AND” gates in T cells is the use of synthetic Notch (synNotch) receptors (26). Sensing of antigen 1 by the synNotch receptor induces transcription of a CAR that is specific for antigen 2 (27) (Figure 3C). Use of both of these strategies to generate antigen-sensing circuits resulted in specific efficacy against tumors with dual antigen expression while sparing target cells expressing either antigen alone. Boolean “OR” as well as “AND” gates offer exciting opportunities to enhance efficacy and precision of tumor targeting. By adding a second antigen specificity, on-target, off-tumor toxicities could potentially be prevented (19). The approach of combinatorial antigen targeting may help to overcome the current challenge of identifying suitable target molecules for CAR T cell therapy for solid tumors. However, this promising preclinical data still needs to be validated in clinical studies and the global adoption of one strategy to all different solid tumor entities seems unlikely. One could picture a scenario where combinatorial antigen approaches are exploited to tailor therapy to the individual patient's characteristics. Taking into consideration the tumor entity and stage, one could employ “OR” gates in cases where enhancing anti-tumor efficiency or preventing antigen escape are essential, while using “AND” gates in cases where on-target, off-tumor toxicity is the major concern.

#### Universal adaptor CAR

In recent years, several research groups have developed platforms for making universal CARs. The general idea is to have an adaptor CAR that binds to a soluble adaptor which in turn conveys specificity against a certain tumor antigen (28, 29, 30, 31, 32) (Figure 3D). This approach allows targeting multiple tumor antigens simultaneously through the combined application of the distinct soluble adaptors and thus is an exciting strategy to address solid tumor heterogeneity. An additional advantage of universal CARs is the ability to redirect the CAR T cell to a new target molecule without having to re-engineer the T cell itself by simply switching the soluble adaptor in case of antigen escape or insufficient tumor response. At the same time, the universal CAR platform implies an “ON-switch” system since the soluble adaptor must be administered for the CAR T cell to be able to become active. This feature provides an additional regulatory element with the possibility to attenuate or abolish CAR T cell function by withdrawing the soluble adaptor or even applying a nonspecific adaptor to compete the specific soluble adaptor off.

However, the clinical feasibility of such universal CAR platforms remains to be evaluated. The complex interaction of the different control features this approach provides will have to be examined individually and jointly. The following factors will have to be explored to optimize clinical outcome: number of adoptively transferred universal CAR T cells, dosage regimen of the soluble adaptor, binding kinetics between the target molecule and the soluble adaptor as well as between the universal CAR T cell and the soluble adaptor. All “ON-switch” CAR T cell platforms entail the additional challenge of deciding when to stop the administration of the CAR activating drug in the case of tumor remission.

## Getting CAR T cells to solid tumors and getting them to stay

Insufficient trafficking to and expansion at the tumor site after systemic administration has been identified as a major hurdle to the success of CAR T cell therapy in solid tumors. The mechanisms governing chemotaxis of T cells to the tumor site and the role of the tumor microenvironment in inhibiting CAR T cell migration and expansion have been comprehensively reviewed recently (33–35). Here we will focus on regional delivery as a means to bypass the necessity of T cells trafficking to the tumor and highlight some innovative engineering approaches to improve T cell persistence.

### Regional delivery

To circumvent the challenge of CAR T cells having to traffic into the tumor, several investigators have focused on regional delivery of CAR T cells for the treatment of solid tumors. Preclinical testing has consistently reported significantly lower CAR T cell numbers being required to induce tumor responses and limited or abolished systemic toxicities when a regional administration route is chosen over systemic delivery (36–38). Mesothelin is expressed on a broad range of solid tumors; lung, pancreatic, breast, and ovarian cancer amongst others, and is under active investigation as a target molecule for CAR T cell therapy. The effects of regional delivery of CAR T cells targeting mesothelin in the context of malignant pleural disease have been studied by Adusumilli and colleagues. In a preclinical model of pleural malignancy, they found that intrapleural injection of mesothelin specific CAR T cells improved T cell activation and persistence as well as tumor response compared to intravenous administration of CAR T cells. Importantly, a significantly lower number of CAR T cells was needed for tumor eradication when administered locally as opposed to systemically. Furthermore, the regionally primed CAR T cells were able to traffic to and clear tumors at distant sites (37). Based on these promising preclinical data, a phase I trial with regionally delivered anti-mesothelin CAR T cells for malignant pleural disease was initiated (NCT02414269). A preliminary report from this study noted no evidence for toxicity while antitumor activity has been observed. CAR T cells could be detected in the peripheral blood of 6 of the 12 patients treated. Encouragingly, one patient, who had additionally received anti-PD1 checkpoint blockade off protocol, achieved a complete remission as evidenced by PET scan (ASGCT 21st Annual Meeting Abstracts Molecular Therapy, Volume 26, Issue 5, 1–459).

Glioblastoma and brain metastasis are solid tumor entities where regional administration of CAR T cell therapy is actively being explored. Preclinical models have shown antitumor efficiency and safety of intracranial administration of EGFRvIII and HER2 redirected CAR T/NK cells (39–41). To date, the clinical outcomes of 5 patients receiving intrathecally or intracranially delivered IL-13Rα2 targeting CAR T cells for glioblastoma have been reported (18, 42, 43). One patient achieved a 7.5 month lasting complete regression of all intracranial and spinal tumors under continued CAR T cell treatment, which is a remarkable occurrence in this disease.

Further phase I clinical trials investigating intratumoral injection of CAR T cells targeting ErbB for the treatment of locally advanced squamous cell cancer of the head and neck (NCT01818323) (44) and hepatic artery infusion of CEA specific CAR T cells combined with SIRT (selective internal radiation therapy) for CEA positive liver metastasis (NCT02416466) are underway.

A whole new approach to regional delivery of CAR T cells for solid tumors using implantable biopolymer scaffolds has recently been reported by Smith et al. (38). The authors showed that regional delivery and expansion of CAR T cells in biopolymer scaffolds implanted at the tumor site in contrast to systemic administration led to superior antitumor responses in mouse models of pancreatic cancer and melanoma. Furthermore, the simultaneous transfer of CAR T cells and stimulator of INF genes agonist by biopolymer scaffold could extend the immune response to tumor cells not expressing the CAR specific target molecule. Another possible advantage of scaffold-assisted delivery may lie in the ability to protect CAR T cells from the hostile influence of the tumor microenvironment by locally supplying them with growth factors during the initial phase of tumor priming.

### Strategies to improve persistence

Longer persistence of CAR T cells posttreatment has been associated with better clinical outcome in both patients with hematological and solid cancers (16, 45, 46). The beneficial effect of prior lymphodepletion, including diminution of regulatory T cells, on CAR T cell engraftment has been established (47–49). Rapid *in vivo* expansion of CAR T cells post infusion, often leading to cytokine release syndrome correlates with anti-tumor responses in hematological malignancies and has been frequently observed in clinical trials using CD19- and BCMA-redirected CARs (50, 51). In contrast, CAR T cell trials for solid tumors have not reported outcomes with strong release of proinflammatory cytokines preceding tumor regression. Therefore, it seems likely that insufficient expansion and persistence of CAR T cells in patients with solid tumors is a major cause for the unsatisfying response rates observed so far. Indeed, insufficient engraftment and persistence of solid tumor specific CAR T cells has been reported in several clinical trials. In a study treating melanoma patients with GD2 specific CAR T cells, only 1 out of 6 patients still had detectable CAR T cells beyond 4 months (52). Monitoring of persistence of anti-EGFRvIII engineered T cells in a trial with r/r glioblastoma patients showed rapid reduction of CAR T cell numbers in peripheral blood starting 2 weeks posttreatment (53).

#### Empowering CAR T cells to shape their own cytokine environment

Cytokine support is a crucial factor for the survival and expansion of T cell therapies. This is particularly true when they encounter hostile conditions as in the microenvironment of solid tumors. Engineering solutions for adoptively transferred T cells have been developed to allow for both, to support themselves with proinflammatory cytokines, and to shield themselves from immunosuppressive cytokines. IL-12 and IL-18 secreting CAR T cells have been shown to persist longer and lead to enhanced tumor responses in preclinical models of solid cancers (54–56). Other investigators have described improved antitumor efficiencies of CAR T cells equipped with constitutive IL-7 and IL-15 signaling, as well as by inducible delivery of IL-15 super-agonist complex by T cells upon encounter of the cognate antigen (57–59).

Taking the reverse approach, the tumor cells' immunosuppressive cytokine signaling can be inhibited or converted into proinflammatory signaling. Overexpression of a dominant negative form of the TFG-β receptor has been reported to increase the anti-tumor potency of CAR T cells against melanoma in a mouse model (60). A phase I clinical trial currently investigates the use of TFG-β resistant CAR T cells directed against PSMA for castrate-resistant prostate cancer (NCT03089203; Table 1). By endowing CAR T cells with an inverted cytokine receptor, consisting of the exodomain of the IL-4 receptor fused to the IL-7 receptor endodomain, signaling of the immunosuppressive cytokine IL-4 could be transformed to promote proliferation and anti-tumor efficiency *in vivo* (61).

Engineering approaches that provide CAR T cells with endogenous cytokine support can be categorized into those where interleukins are secreted into the surroundings and those where interleukin signaling is restricted to the CAR T cell itself. Besides providing autocrine stimulation for the CAR T cell itself, secreting approaches may have additional paracrine effects e.g., remodeling the tumor microenvironment and activating by-stander immune cells (55). Yet they come at the risk of causing systemic inflammatory reactions and toxicities, as have been previously reported upon systemic cytokine administration (62). Koneru and colleagues therefore carefully designed their phase I clinical trial of IL-12 secreting MUC-16(ecto) targeting CAR T cells for the treatment of recurrent ovarian cancer by adding an “off-switch” (tEGFR) and administering half the CAR T cell dose intraperitoneally in order to enhance safety (NCT02498912; Table 1) (63).

#### Targeted CAR integration into the T cell genome

We have learned from hypothesis driven research and clinical observation that the genomic integration site of the CAR fundamentally impacts the T cell's ability to activate and persist. Targeted insertion of the CAR into the TRAC locus, as opposed to random insertion during conventional CAR T cell manufacturing, enhanced the T cells anti-tumor function in a leukemia mouse model. Delivery of the CAR into the TRAC locus prevented functional exhaustion of the T cells by circumventing tonic CAR signaling, i.e., activation in the absence of the cognate antigen (64).

Fraietta and colleagues recently reported the case of a patient in which the clonal expansion of one single CAR T cell induced remission of chronic lymphocytic leukemia. Further analysis revealed that random insertion of the CAR into the TET2 gene locus had led to disruption of TET2 protein expression in this patient who also had a hypomorphic mutation on their other TET2 allele; the biallelic disruption of TET2 resulted in a central memory state of the T cell clone (65).

Since functional exhaustion and insufficient expansion of T cells have been identified as major shortcomings of CAR T cell therapy for solid cancers, these innovative strategies may help balance some of the challenges encountered. Targeted CAR delivery into the T cell genome holds promise to generate phenotypically more competent cells and thereby enhance their anti-tumor efficiency. However, further research is needed in order to determine feasibility and safety of directed CAR delivery into the T cell genome.

#### Preventing *ex vivo* differentiation and exhaustion of CAR T cells

The current CAR T cell manufacturing process requires *ex vivo* activation and expansion of the patient's T cells. This may speed up effector T cell differentiation and functional exhaustion, thereby reducing the potency of the CAR T cell product.

The use of T cell homing nanoparticles has recently been suggested as a new approach to CAR T cell production. T cell homing nanoparticles can reprogram T cells *in vivo*, without the need to remove them from the subject's body. After administration, the nanoparticles deliver CAR encoding DNA selectively to T cells. *In vivo* reprogrammed CAR T cells were as efficient as *ex vivo* manufactured conventional CAR T cells at controlling leukemia progression in a preclinical mouse model (66). The current standard CAR T cell manufacturing protocols requiring *ex vivo* engineering of the adoptive cell product cause time delays, high costs and potentially have a negative impact on the T cell phenotype. However, one of its strength is the long safety record in clinical application. Long-term follow up of patients treated with retroviral engineered CAR T cells has not shown any transformational events in more than 500 patient-years of follow up (67). *In vivo* administration of CAR delivering nanoparticles comes with the risk of unintentional gene transfer into off-target cells. Accidental gene transfer into hematopoietic stem cells represents a major safety concern, since malignant transformation of hematopoietic stem cells causing leukemia has previously occurred in pioneering gene therapy trials (68, 69). Further research is needed to establish the safety profile of gene delivering nanoparticles before they can be translated into clinical application for *in vivo* CAR T cell manufacturing.

Other strategies to avert the negative impact of *ex vivo* culture on the CAR T cells antitumor potency is to make sure CAR signaling starts only post-infusion of the product. The concept of a CAR integrated “ON-switch” was introduced by Wu et al. (70). They designed a split CAR where the functional components of a conventional CAR are dissociated into two parts that only reassemble in the presence of a small molecule. The Tet-OFF CAR platform proposed by Mamonkin and colleagues employs a conditional doxycycline regulated system where the CAR is only expressed upon withdrawal of the drug (71). Both these strategies permit to switch on the CAR expression only post transferal of the adoptive T cell therapy. “ON-switch” concepts for CAR expression combine the advantages of maintaining a more naïve T cell phenotype with the distinguished safety features of *ex vivo* genetic engineering of T cells; thus, for now their clinical translation seems more feasible than *in vivo* CAR T cell engineering.

## Conclusions

CAR T cells for the treatment of solid tumors have made progress for individual target antigens and tumor entities. Broader proof of concept for the efficiency of immunotherapy in solid cancers has been provided by the considerable success of checkpoint blockade. As a “living drug” CAR T cell therapies confer the advantage of potentially life-long tumor surveillance. Lessons learned from the unsatisfying response rates of most pioneering CAR T cell trials for solid tumors have fed back into preclinical development of new concepts to address these hurdles. CAR T cells for solid tumors have passed through the first cycle, form bench to bedside and back. Still there is need for considerable optimization before CAR T cell therapy can advance as a standard treatment option for patients with solid tumors. However, the emerging preclinical and clinical research on identifying suited target antigens as well as improving delivery and persistence of CAR T cells in solid cancer holds promise for wider therapeutic applications.

## Author contributions

AS and MM wrote the manuscript and created the figures. Both authors edited the manuscript and approved it for publication.

### Conflict of interest statement

The authors declare that the research was conducted in the absence of any commercial or financial relationships that could be construed as a potential conflict of interest. The reviewer BO and handling Editor declared their shared affiliation.
